# Atypical Asparagine Deamidation of NW Motif Significantly Attenuates the Biological Activities of an Antibody Drug Conjugate

**DOI:** 10.3390/antib12040068

**Published:** 2023-10-24

**Authors:** Mingyan Cao, G. Patrick Hussmann, Yeqing Tao, Ellen O’Connor, Conner Parthemore, Diana Zhang-Hulsey, Dengfeng Liu, Yang Jiao, Niluka de Mel, Meagan Prophet, Samuel Korman, Jaytee Sonawane, Christina Grigoriadou, Yue Huang, Scott Umlauf, Xiaoyu Chen

**Affiliations:** 1 Department of Process and Analytical Sciences, Biopharmaceutical Development, BioPharmaceuticals R&D, AstraZeneca, One Medimmune Way, Gaithersburg, MD 20878, USA; mingyan.x.cao@gsk.com (M.C.); jiaoyang88552@gmail.com (Y.J.); niluka.demel@astrazeneca.com (N.d.M.); christina.grigoriadou@astrazeneca.com (C.G.);; 2 Department of Integrated Bioanalysis, Clinical Pharmacology & Safety Sciences, R&D, AstraZeneca, 121 Oyster Point Boulevard, South San Francisco, CA 94080, USA

**Keywords:** deamidation, post-translational modification (PTM), antibody drug conjugate (ADC), critical quality attributes (CQAs), biological activity

## Abstract

Asparagine deamidation is a post-translational modification (PTM) that converts asparagine residues into iso-aspartate and/or aspartate. Non-enzymatic asparagine deamidation is observed frequently during the manufacturing, processing, and/or storage of biotherapeutic proteins. Depending on the site of deamidation, this PTM can significantly impact the therapeutic’s potency, stability, and/or immunogenicity. Thus, deamidation is routinely monitored as a potential critical quality attribute. The initial evaluation of an asparagine’s potential to deamidate begins with identifying sequence liabilities, in which the n + 1 amino acid is of particular interest. NW is one motif that occurs frequently within the complementarity-determining region (CDR) of therapeutic antibodies, but according to the published literature, has a very low risk of deamidating. Here we report an unusual case of this NW motif readily deamidating within the CDR of an antibody drug conjugate (ADC), which greatly impacts the ADC’s biological activities. Furthermore, this NW motif solely deamidates into iso-aspartate, rather than the typical mixture of iso-aspartate and aspartate. Interestingly, biological activities are more severely impacted by the conversion of asparagine into iso-aspartate via deamidation than by conversion into aspartate via mutagenesis. Here, we detail the discovery of this unusual NW deamidation occurrence, characterize its impact on biological activities, and utilize structural data and modeling to explain why conversion to iso-aspartate is favored and impacts biological activities more severely.

## 1. Introduction

The market for biopharmaceuticals has grown exponentially over the past 2–3 decades, leading to the treatment and prevention of a vast range of diseases and disorders. In parallel, more sophisticated biopharmaceuticals are emerging on the market, including antibody drug conjugates and cell and gene therapies. This growth in biologics is, in part, due to their continued demonstration of high specificity, thereby enabling more targeted actions with fewer and/or less severe adverse events. Moreover, the efficiency of manufacturing biopharmaceuticals has improved greatly over the years, which has ensured more streamlined development, consistent products, and decreased cost.

Nonetheless, manufacturing and control challenges persist, even for simple biopharmaceuticals. For example, protein-based biotherapeutics are prone to numerous chemical and biochemical post-translational modifications (PTMs) during manufacturing, processing, and storage. These PTMs—including glycosylation, glycation, oxidation, and deamidation—can impact the stability, potency, pharmacokinetics, and/or immunogenicity of the product, thereby compromising clinical efficacy and safety [1].

Asparagine deamidation is one PTM that converts asparagine residues into iso-aspartate and/or aspartate [2,3]. The converted aspartate and iso-aspartate residues are largely of the L-isomer; however, D-isomers have been detected at low levels following deamidation [4]. Deamidation can occur both enzymatically [5] and non-enzymatically [6,7,8]. The susceptibility and rate of non-enzymatic deamidation is dependent on many variables, including pH, temperature, solvent/buffer and excipients, and physical state (i.e., liquid vs. lyophilized) [2,9]. Protein primary structure, as well as secondary and tertiary structure, also influence the susceptibility and rate of protein deamidation [10,11,12,13].

Deamidation has a high potential to impact protein structure and function, as it induces a change in residue charge, hydrophobicity, and mass. In nature, deamidation is ubiquitously and frequently observed, leading some to hypothesize that endogenous deamidation is a mechanism for regulating physiological processes. For instance, some claim that non-enzymatic deamidation regulates protein turnover, thereby acting as a biomolecular clock, in accordance with deamidation rates, to regulate organism development and aging [14,15]. Others report deamidation as a switch for apoptosis following DNA damage [16], or even a tool for pathogens to evade the innate immune system [17].

As in nature, deamidation is observed frequently in protein-based therapeutics. Depending on the location of the deamidated site, this PTM has the potential to significantly impact the stability, potency, pharmacokinetics, and/or clinical safety of biotherapeutics. For example, Lu et al. report that deamidation of the antibody variable fragment (Fv) of Moxetumomab pasudotox results in impaired target binding, internalization, and delivery of cytotoxic payload to cancer cells [18]. As another example, deamidation in the crystallizable fragment (Fc) region of an anonymized therapeutic antibody was reported to decrease Fc effector functions through decreased FcyRIIIa binding [19]. Hence, deamidation is routinely monitored as a potential critical quality attribute (pCQA) in the biopharmaceutical industry.

CQA evaluations for deamidation usually begin with the identification of relevant amino acid sequence liabilities, as, again, the primary structure is known to influence deamidation susceptibility. The amino acid in the n + 1 position is of particular interest when identifying liabilities for asparagine deamidation. Amino acid motifs NG, NS, NN, NG, and NH are commonly found within the complementarity-determining region (CDR) of antibodies and are considered canonical for deamidation, in which NG is more labile than NS, and NS more labile than NT or NN and NH. On the other hand, the common CDR motifs NY, NW, NQ, and NF are considered non-canonical, with a low risk for deamidation [20].

ADC-A is an anonymized antibody drug conjugate (ADC) that was under recent development for cancer indication. ADC-A is composed of a monoclonal antibody (mAb) intermediate conjugated to a cytotoxic small molecule warhead via a chemical linker. The cytotoxic warhead is specifically conjugated at an inserted cysteine site in the hinge region of each mAb intermediate heavy chain (HC) [21,22], which allows tight control of a targeted drug-to-antibody ratio (DAR) of two. The primary mechanism of action of ADC-A is warhead-induced cancer cell death, which is initiated via the ADC-A binding to its target antigen, expressed on the surface of cancer cells. Once bound, the ADC-A-antigen complex is internalized within the cancer cell and degraded through lysosomal activity, thereby freeing the cytotoxic warhead to induce cell death. The specific warhead of ADC-A elicits cytotoxicity by binding and crosslinking DNA, thereby inhibiting transcription and replication processes.

The initial analysis of sequence liabilities for this mAb intermediate identified an N102–W103 motif in the CDR. Observing the NW motif itself was not surprising nor a concern, as again NW is found frequently within the CDR of antibodies, but demonstrates low risk for deamidation [12,13,20]. In fact, in one study that evaluated over 100 clinical-stage therapeutic antibodies, NW was identified as the second most commonly occurring asparagine motif within CDRs but demonstrated the lowest rate of deamidation among all canonical and non-canonical motifs [20]. Therefore, N102–W103 was not deemed a sequence liability. However, during later forced-degradation/CQA evaluation studies, ADC-A and its mAb intermediate were found to readily deamidate at N102. Moreover, N102 deamidation was observed not only under forced degradation conditions, but also during normal manufacturing process conditions, resulting in up to approximately 15–20% of mAb intermediate product being deamidated at this residue.

Further investigations of mAb intermediate/ADC-A deamidation revealed that N102 deamidation converts the asparagine residue into nearly 100% iso-aspartate. This finding is strikingly different from previous reports, where a mixture of aspartate and iso-aspartate is typically observed following deamidation through a succinimide intermediate [2,3]. Regarding biological activity, N102 deamidation significantly attenuates the binding affinity of the mAb intermediate to the target antigen. This impact on target binding persists after payload conjugation, which consequentially impairs ADC-A’s ability to internalize into tumor cells and induce a cytotoxic effect in vitro. Interestingly, the biological activity of ADC-A is more severely reduced when N102 is deamidated into isoD102, as compared to when N102 is mutated to N102D. Here, we report the discovery of this atypical NW deamidation, characterize its impact on biological activity, and use structural data and modeling to explain the differential impact of isoD102 versus N102D on activity.

## 2. Materials and Methods

### 2.1. Reagents and Materials

Engineered antibody intermediate and antibody drug conjugates were produced by AstraZeneca (Gaithersburg, MD, USA). Synthetic peptides were obtained from Tufts University (Boston, MA, USA). HepG2 and T47D cells were obtained from ATCC (Gaithersburg, MD, USA). Dulbecco’s Modified Eagle Medium (DMEM), Advanced DMEM, MEM non-essential amino acids (NEAA), and GlutaMax™ were obtained from Thermo Fisher Scientific (Waltham, MA, USA). Ammonium sulfate, sodium acetate N-ethylmaleimide, sodium phosphate dibasic, sodium phosphate monobasic monohydrate, sodium chloride, formic acid, trifluoroacetic acid (TFA), Fetal Bovine Serum (FBS), and 10× Phosphate Buffered Saline (PBS) pH 7.4 were obtained from Sigma-Aldrich (St. Louis, MO, USA). Urea (OmniPur), water (OmniSolv, HPLC and spectrophotometry grade), acetonitrile (OmniSolv, HPLC and spectrophotometry grade), and isopropyl alcohol (OmniSolv, HPLC and spectrophotometry grade) were obtained from EMD Serono (Rockland, MA, USA). LysC and CellTiter-Glo^®^ were obtained from Promega (Madison, WI, USA). Dithiothreitol (no-weight format) was obtained from Pierce Protein Biology (Waltham, MA, USA). All 96-well microplates were obtained from VWR (Radnor, PA, USA). DELFIA^®^ Eu-N1 anti-human IgG, DELFIA^®^ assay buffer, DELFIA^®^ wash buffer, and DELFIA^®^ enhancement solution were obtained from PerkinElmer (Waltham, MA, USA). Incucyte^®^ Human Fabfluor-pH Red antibody labeling dye was obtained from Sartorious (Göttingen, Germany).

### 2.2. Purification of Non-Deamidated and Single-Deamidated Species

ADC-A contained a mixture of double deamidated, single deamidated, and non-deamidated species. Separation of these materials was performed using Source 15S cation exchange resin (Cytiva, Marlborough, MA, USA). The resin was packed into a 2.2 cm × 20 cm Vantage Column (MilliporeSigma, Burlington, MA, USA) which was operated at 150 cm/h on an Akta Avant (Cytiva, Marlborough, MA, USA). Prior to separation, the resin was sanitized with 1 N sodium hydroxide and then equilibrated with 25 mM acetate pH 5.0. ADC-A was applied to the equilibrated resin at 20 g/L. The loaded resin was re-equilibrated prior to a 20 column-volume gradient elution to 25 mM acetate and 500 mM sodium chloride, at pH 5.0. Fractions were collected during elution and analyzed for protein concentration, purity, and deamidation. As expected, the material eluted from the column according to charge, with double deamidated species eluting prior to the single and non-deamidated materials. There were 60 mg of single deamidated product and 300 mg of non-deamidated product obtained, both with monomeric purities greater than 99.5%. All chemicals were JTBaker USP grade (VWR, Radnor, PA, USA).

### 2.3. Non-Deamidated, Deamidated, and N102D Mutant mAb Intermediate Conformations Were Compared by Hydrogen–Deuterium Exchange (HDX)

Deamidated (isoD-form) mAb intermediate, collected by fractionation, and N102D mutant (D-form) were compared with the non-deamidated protein (N-form), using HDX, to investigate the protein conformational changes caused by deamidation or mutation around the PTM/mutation site or globally. The HDX experimental procedure was fully automated and conducted by Waters HDX Manager (Waters, Milford, MA, USA) with the temperature-controlled LEAP liquid handling system (Trajan, Morrisville, NC, USA). Two HDX experiments were performed: non-deamidated (N form) vs. deamidated (isoD-form), and non-deamidated (N form) vs. N102D mutant (D form). To initiate HDX, 3 µL of the sample, at approximately 10 mg/mL, was diluted tenfold with 50 mM sodium phosphate buffer at pH 7.0 in D2O. The exchange reaction was temperature controlled at 24 °C for 0.5, 1.5, 5, 15, and 60 min, in triplicates. The exchange reaction was quenched by adding the same volume of pre-chilled acidic quenching buffer, composed of 200 mM glycine, 8 M guanidine HCl, and 500 mM TCEP at pH 2.5 under 0 °C. The quenched samples were then diluted fourfold with 0.1% formic acid (pH 2.5, 0 °C) and injected onto the digestion column inside the Waters HDX manager with the cooling chamber at 0 °C. The samples were on-column digested by passing through a column with immobilized pepsin resin (Waters, 2.1 × 30 mm). The digested peptides were desalted on a VanGuard precolumn (Waters, 2.1 × 5 mm) for 2.5 min at a flow rate of 100 μL/min, and subsequently separated in an analytical column (Waters Acquity BEH300 column, 1.0 × 100 mm) at a flow rate of 40 μL/min. The automation method was created and controlled by Chronos software version 2.1 (Trajan Automation, Morrisville, NC, USA). Experiments were undertaken in triplicates for each time point.

### 2.4. Liquid Chromatography–Mass Spectrometry (LC–MS) Analysis of HDX Samples

The digested peptides were separated with a 6.8 min gradient of 15% to 28% Solvent B (Solvent B: acetonitrile with 0.1% formic acid; Solvent A, H_2_O with 0.1% formic acid). For N-form vs. isoD-form comparison, data were acquired on a QExactive HF-X mass spectrometer (Thermo Fisher Scientific, Waltham, MA, USA); and for the N-form vs. D-form experiment, the data were collected on Eclipse (Thermo Fisher Scientific, Waltham, MA, USA). Peptide digestion without D_2_O was undertaken on QE-HF and Eclipse, with the precursor ions mass range at 300–2000 *m*/*z* for all samples, with a 120,000 resolving power. From each MS scan, the top ten most abundant precursors were selected for fragmentation by collision-induced dissociation (CID) in a data-dependent acquisition (DDA) mode, with a fixed collisional energy of 27%. Deuterium labeling data were collected similarly without MS/MS scans. Xcalibur 2.2 (Waltham, MA, USA) was employed for data acquisition.

### 2.5. HDX Data Analysis

MS data in the digestion runs were analyzed by Byos to identified all the peptides. (Protein Metrics, San Carlos, CA, USA). The deconvoluted peptide masses were searched against the non-specific digestion of amino acid sequences of the mAb intermediate or the mAb intermediate with N102D. The search tolerance window was set to 10 ppm mass error for precursor ions and 50 ppm mass error for product ions. The identified peptide list was imported into HD Examiner software version 2.1 (Sierra Analytics, Modesto, CA, USA) to calculate the numbers of deuterium uptake (D-uptake) of each peptide at each time point and later compared between the N-form vs. isoD-form and N-form vs. D-form, to make the HDX differential plots.

Homology models for the N-form, isoD-form, and D-form were generated using an antibody modeler by Molecular Operating Environment (MOE). The MOE modeler in this study used Protein Data Bank (PDB) to build the homology models. In Fab homology modeling, mAb intermediate N-form and D-form sequences were searched against the database and templates with the highest similarity for the framework region sequences and CDR loop sequences were identified. The modeler then grafted CDR loop templates onto the light chain or heavy chain frameworks. The transition areas between CDRs and frameworks were fine-tuned by energy minimization using an AMBER99 force field. HDX data were visualized by the HDX module of MOE. The color code scheme “Jet” was applied to illustrate the D-uptake differences between the N-, isoD- and the D-forms.

### 2.6. Analytical CEX

The identity of each fraction was verified by analytical scale CEX analysis. Briefly, 75 μL of each sample and Reference Standard diluted to 1.0 mg/mL in mobile phase A were injected onto a Dionex Propac™ WCX-10 ion exchange column (4 × 250 mm). The mobile phases used were 20 mM sodium phosphate at pH 7.0 for mobile phase A and 20 mM sodium acetate containing 250 mM sodium chloride at pH 7.0 for mobile phase B. A gradient of 35.0 to 95.0% mobile phase B was run from 5 to 55 min at a flow rate of 1 mL/min. The eluted protein was detected using UV absorbance at 220 nm.

### 2.7. DAR Measurement by RP-HPLC

The ADC-A or mAb intermediate samples were made to 2 mg/mL in HPLC water. To each sample with 50 μL (100 μg), 50 μL denaturing buffer (8 M guanidine HCl, 160 mM Tris, 1 mM EDTA, pH 7.6) and 2 μL of 500 mM DTT (dithiothreitol) were added and the mixture was incubated at 37 °C for 30 min. From each sample, 10 μL was loaded onto a Waters BioResolve RP mAb polyphenyl column (2.1 × 150 mm, 2.7 µm, 450Å). Mobile phase A and B consisted of 0.1% TFA in water and 0.1% TFA in acetonitrile, respectively. A chromatography method with a linear gradient of 32.5% to 46.5% mobile phase B from 2 to 30 min at a flow rate of 0.5 mL/min was adopted, with the eluted protein detected at a UV wavelength of 280 nm. DAR and drug-load distribution were calculated based on peak areas.

### 2.8. Reduced Peptide Mapping

Ten µL of protein samples at 10 mg/mL were denatured, reduced, and alkylated. The denaturation and reduction were performed using 20 µL denaturing buffer (7.9 M Guanidine-HCl, 100 mM Tris, 0.1 mM EDTA, pH 7.3–7.4) and 2 µL DTT at 500 mM, incubated at 37 °C for 30 min. The alkylation was conducted by adding 5 µL 500 mM IAM (iodoacetamide) and incubated at room temperature for 30 min in the dark. Samples (volume ~37 µL) were then transferred to a Pierce™ 96-well Microdialysis Plate, 10 K MWCO (Thermo, Part # 88260), which were pre-conditioned with 1400 µL dialysis buffer (6 M urea, 150 mM Tris, pH 7.5). Dialysis was performed using a mixer at 500 rpm for 2 h, at room temperature in dark. After dialysis, the protein solution was recovered to Eppendorf tubes and 70 µL of digestion buffer (100 mM Tris, pH 7.5) was added. Half of the mixed solution (volume ~50 µL) was aliquoted for trypsin digestion, with the protein enzyme ratio being kept at 12.5:1. This was digested for 3.5 to 4 h at 37 °C and quenched by using *v*/*v* 2% TFA. The resulting solution contained approximately 1 mg/mL protein (tryptic digests), 2 M urea, and 2% TFA. Finally, the peptide fragments, generated by the enzymatic cleavage of ADC-A by trypsin, were separated using UPLC and analyzed by UV detection followed by tandem mass spectrometry. The peptide mapping analysis with LC–MS was performed using a Waters Acquity UPLC system and a Waters Acquity C18 BEH300, (1.7 µm, 2.1 × 150 mm) with mobile phase A and B, which consisted of 0.02% TFA in HPLC water and 0.02% TFA in acetonitrile, respectively, and a Thermo LTQ Orbitrap mass spectrometer.

### 2.9. Cell-Based Binding Assay

HepG2 cells, which endogenously express ADC-A’s target antigen at the cell surface, were prepared in cell culture media (DMEM, 10% FBS, and 1× MEM NEAA) and seeded in Corning^®^ 96-well PureCoat™ amine plates at 1.5 × 10^5^ cells/well. Plates were then incubated at 37 °C under 5% CO_2_ in a humified incubator overnight. The following day, plates were washed with 1× PBS pH 7.4 using a BioTek 405 LS plate washer. Increasing concentrations of ADC-A Reference Standard and test samples (24,000–0.68 ng/mL) were then added to the plate wells, and the plates were incubated at an ambient temperature for 40–60 min. Following incubation, the plates were washed with 1× PBS pH 7.4. A 65 ng/mL solution of DELFIA^®^ Eu-N1 anti-human IgG prepared in DELFIA^®^ assay buffer was then added to the plate wells, and the plates incubated at ambient temperature for 50–70 min. Following incubation, the plates were washed with 1× DELFIA^®^ wash buffer. DELFIA^®^ Enhancement solution was then added to the plate wells, and the plates were incubated for 15–60 min while being gently shaken at an ambient temperature. Bound DELFIA Eu-N1 anti-human IgG was measured via time-resolved fluorescence using a PerkinElmer Envision microplate reader (PerkinElmer; Waltham, MA, USA). The resulting relative fluorescence units (RFUs) were plotted against the ADC-A concentration using Prism version 9 (GraphPad Software; San Diego, CA, USA), and the data were fit using a 4PL curve fit. EC_50_ values were obtained from the 4PL curve fits. To calculate the %RP, the EC_50_ value of the Reference Standard was divided by the EC_50_ value of the test sample and multiplied by 100. Statistical analyses were performed in Prism 9 (Boston, MA, USA).

### 2.10. Cytotoxicity Assay

T47D cells, which endogenously expresses ADC-A’s target antigen, were prepared in cell culture media (Advanced DMEM, 5% FBS, and 1× GlutaMAX™) and seeded in Corning^®^ 96-well white tissue culture-treated microplates. ADC-A Reference Standard and test samples were also prepared in cell culture media and added to the plate wells at final concentrations of 14,000–5.2 ng/mL. The plates were then incubated at 37 °C under 5% CO_2_ in a humidified incubator for 4 days. Following incubation, an equivalent volume of CellTiter-Glo^®^ luminescent cell viability substrate was added to the plate wells, and the plates were incubated at an ambient temperature while being gently shaken for 20–40 min. Luminescence within the plate wells was then measured with a PerkinElmer Envision microplate reader. The resulting relative luminesce units (RLUs) were plotted against ADC-A concentration using Prism 9, and the data were fit using a 4PL curve fit. IC_50_ values were obtained from the 4PL curve fits. To calculate %RP, the IC_50_ value of the Reference Standard was divided by the IC_50_ value of the test sample and multiplied by 100%. Statistical analyses were performed in Prism 9.

### 2.11. Cell-Based ADC-A Internalization Assay

T47D cells were prepared in cell culture media (Advanced DMEM, 5% FBS, and 1× GlutaMAX™) and seeded in 96-well clear tissue culture-treated microplates at 2 × 10^4^ cells/well. The plates were then incubated at 37 °C under 5% CO_2_ in a humified incubator overnight. The following day, serial dilutions of ADC-A were prepared in Advanced DMEM and incubated with 5 µg/mL Incucyte^®^ Human Fabfluor-pH Red antibody labeling dye for 15–20 min at an ambient temperature. This mixture was then added to plate wells at a final ADC-A concentration of 2.0 µg/mL. Plates were immediately placed into an Incucyte S3 high-content fluorescent imager (Sartorious; Göttingen, Germany) and imaged for phase-contrast and red fluorescence under 20× objective every 20 min. Images were analyzed using Incucyte S3 software version 2020c rev1 (Sartorius, Göttingen, Germany). The confluency of T47D cells was detected within phase contrast images with a segmentation adjustment value of 0.5 and a minimum area filter set at 200 µm^2^. Red fluorescence was detected via top hat segmentation with the radius set at 10 µm and the threshold set at 0.3 RCU. The total red fluorescence area (µm^2^/well) was plotted against incubation time in GraphPad Prism 9. Statistical analyses were performed in Prism 9.

## 3. Results

### 3.1. Correlation of N102 Deamidation, CEX Acidic Peaks and Potency Loss in ADC-A and mAb Intermediate

**Figure 1:**
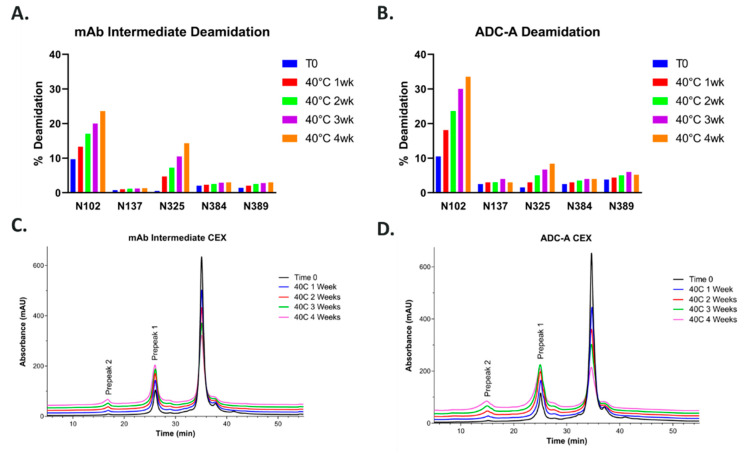
Deamidation sites and levels in ADC-A and mAb intermediate under 40 °C thermal stress conditions and their CEX charge profiles. (**A**) Deamidation in mAb intermediate; (**B**) deamidation in ADC-A; (**C**) CEX profiles of mAb intermediate; (**D**) CEX profiles of ADC-A.

**Figure 2:**
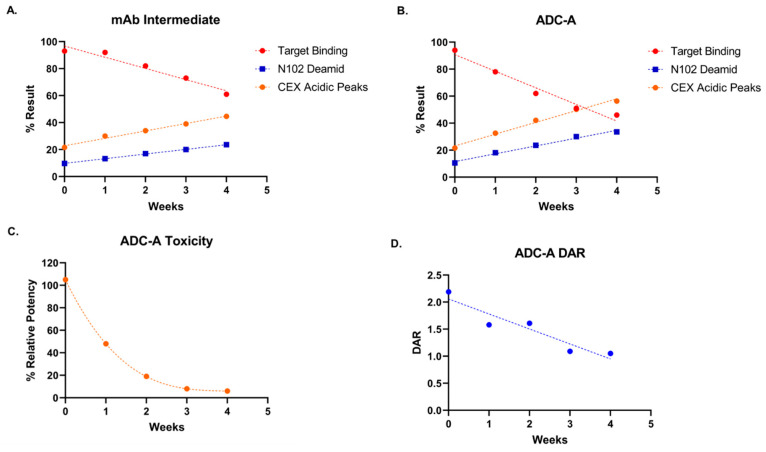
Correlation of N102 deamidation, CEX acidic peak levels, and target antigen binding for (**A**) mAb intermediate and (**B**) ADC-A following 40 °C thermal stress for increasing time. Target binding data are plotted from n = 1 and fit with linear regression. (**C**) %RP of cytotoxicity activity following stress at 40 °C for increasing time. Data are plotted from n = 2 and fit with a non-linear, one-phase decay fit. %RP values at 3- and 4-week thermal stress are estimates, as the 4PL curve fits lacked defined effective asymptotes. (**D**) ADC-A DAR measurements following stress at 40 °C for increasing time. Data are plotted from n = 1 and fit with linear regression.

ADC-A is composed of mAb intermediate, which possesses an inserted cysteine residue in each heavy chain (HC), resulting in a total of two additional cysteines in the hinge region and a targeted DAR of two [21,22]. During development, extensive characterization of ADC-A/mAb intermediate was performed, including forced degradation and weak cation exchange chromatography (CEX) fractionation studies, to identify the degradation pathways, assess structure–function relationships, and confirm the CQAs. In one forced degradation study, ADC-A and mAb intermediate were stressed at 40 °C in formulation buffer for 1, 2, 3 and 4 weeks. Peptide mapping analysis showed that among all the deamidations sites, deamidation at N102 in HC-CDR3 was the predominant deamidation site for mAb intermediate and ADC-A following thermal stress (Figure 1A,B). In CEX analysis, asparagine deamidation created negatively charged aspartate or iso-aspartate, which were more acidic than the main peak and eluted as pre-peaks in CEX (Figure 1C,D). A strong correlation was found between the acidic species in CEX and the N102 deamidation observed in peptide mapping, as both increased at similar rates (Figure 2A,B). Being the predominate deamidation site identified by peptide mapping, N102 deamidation was expected to be the major modification contributing to the increasing acidic variants under these conditions.

Next, thermally stressed ADC-A and mAb intermediate samples were tested for biological activity via two assays: (1) a cell-based target antigen binding assay and (2) a cell-based cytotoxicity assay (for ADC-A samples only). Unstressed mAb intermediate and ADC-A Reference Standards were also tested in these assays side-by-side as comparators for calculating percent relative potency (%RP) values of the stressed samples (see Section 2 for more details).

The results show that the binding activity of mAb intermediate (Figure 2A) and ADC-A (Figure 2B) decreased with increasing N102 deamidation. Moreover, the trend of decreased binding was more pronounced for ADC-A than mAb intermediate, which corresponds with higher rates of N102 deamidation within ADC-A than mAb intermediate under the same stressed conditions (Figure 1). Consistent with ADC-A target binding, the cytotoxicity of ADC-A decreased with increasing N102 deamidation (Figure 2C), but with more severity. Furthermore, the trend in decreased cytotoxicity did not appear linear. The greater impact of thermal stress on ADC-A cytotoxicity than binding is likely attributed to the combination of decreasing target antigen binding affinity along with a decreasing drug-to-antibody ratio (DAR) (Figure 2D). Overall, these data demonstrate a clear correlation between N102 deamidation and reduced biological activities of ADC-A and its mAb intermediate. However, the confounding impact of DAR loss, as well as potential changes to other quality attributes under thermal stress, warranted other strategies to enrich N102 deamidation for further characterization of this phenomenon.

### 3.2. Identification of CDR N102 Deamidation as a CQA for ADC-A

**Figure 3:**
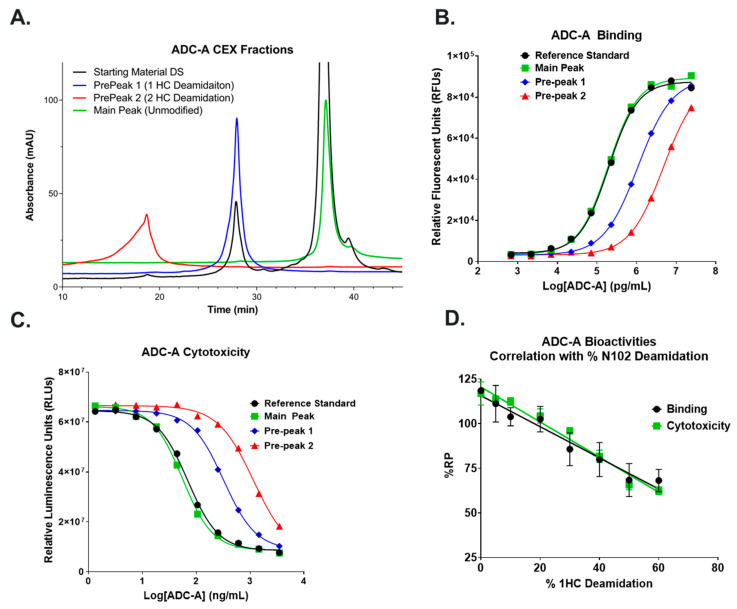
(**A**) CEX fractionation of ADC-A resulted in defined main peak, pre-peak 1, and pre-peak 2. ADC-A factions were tested for (**B**) binding and (**C**) cytotoxicity. Data plotted are from a single experiment performed in triplicate. (**D**) Correlation between increasing percentage of deamidated ADC-A and biological activities. Data are plotted as the mean ± SD from two independent experiments for binding and three independent experiments for cytotoxicity. Data are fitted with linear regression.

In lieu of using thermal stress, CEX was used to enrich ADC-A N102 deamidation through fractionation. Within this CEX fractionation study, pre-peak 1, pre-peak 2 and the main peak were collected from a non-stressed ADC-A drug substance (Figure 3A). Peptide mapping data showed N102 deamidation was enriched to 49% in pre-peak 1 (consistent with deamidation on a single HC) and 65% in pre-peak 2 (consistent with high level of double deamidation), and that deamidation in the Fc region was unenriched (Table 1). Test results from DAR, high molecular species (HMWs), and low molecular species (LMWs) in pre-peak 1 and the main peak were comparable (Supplementary Table S1).

The collected ADC-A pre-peak 1, pre-peak 2, and main peak CEX fractions were then tested for target antigen binding (Figure 3B) and cytotoxicity (Figure 3C). Unfractionated ADC-A Reference Standard, which possessed ~15% deamidation, was also tested side-by-side as a comparator. The main peak fraction bound the target antigen with 102% RP and induced cytotoxicity with 126% RP, compared to the Reference Standard. Pre-peak 1 fraction bound the target antigen with 18% RP and induced cytotoxicity with 22% RP. These results demonstrate that N102 deamidation in just one HC significantly impacts ADC-A’s biological activities. Pre-peak 2 fraction bound the target antigen and induced cytotoxicity with even less potency; however, numerical values could not be accurately calculated due to the lack of defined asymptotes in the 4-parameter logistic (4PL) curve fits that correspond to maximum effect (E_max_), and thus parallelism between curves could not be established. Pre-peak 2′s further reduction in biological activity, as compared to pre-peak 1, correlates with pre-peak 2 possessing more N102 deamidation at both HCs.

To further characterize the impact of N102 deamidation on biological activities, ADC-A pre-peak 1 fraction was spiked into the main peak fraction at increasing levels and tested for binding and cytotoxicity. The resulting %RP values were then plotted against % ADC-A deamidation, and the data were fitted with linear regressions (Figure 3D). The linear regressions showed a strong correlation of decreasing %RP for binding and cytotoxicity with increasing percentage of deaminated ADC-A at 1 HC. Specifically, the slopes of the linear fits were −0.87 and −0.98 for binding and cytotoxicity, respectively, with Pearson correlation coefficient (r) values of −0.98 and −0.99. Overall, these results confirm that increased N102 deamidation significantly impacted ADC-A biological activities in a linear manner.

### 3.3. Comparison of NW Deamidation Products and Deamidation Rates in ADC-A, mAb Intermediate, and Synthetic Peptides

Deamidation of asparagine could happen via the three degradation pathways: (1) nucleophilic attack of the alpha-NH group on the carbonyl carbon of the side chain, leading to a five-membered succinimide (cyclic imide) intermediate that hydrolyzes into a mixture of aspartate and iso-aspartate; (2) nucleophilic attack by the backbone carbonyl oxygen on the carbonyl carbon of the side chain, resulting in a cyclic isoimide intermediate, yielding only aspartate after hydrolysis; or (3) asparagine residues deamidating to aspartic acid by direct water-assisted hydrolysis [2,12,20].

To identify NW degradation products in ADC-A, and to assess the contribution of primary structure to NW degradation, the following synthetic peptides were obtained: N102 (identical to the native tryptic peptide in ADC-A), its D102 form, and isoD102 form. In addition, three short peptides (S. Peptides) were obtained. S. Peptide 1 contains the same sequence as peptide N102 but has only eight amino acids. S. Peptide 2 and 3 contain a substitution at n + 2 (H to V) and n − 1 (K to V), respectively, compared with S. Peptide 1. The hydrophobic V residue was selected as a substitute, so as to assess the impact of H and/or K’s charge on the NW deamidation rate, through possible interference or enhancement with the 5-membered succinimide ring formation and hydrolysis. S. Peptide sequence information is shown in Table 2.

**Figure 4:**
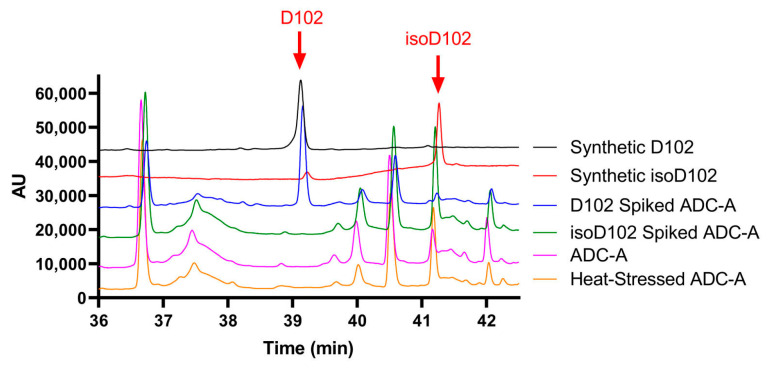
RP–LC–MS UV profiles of synthetic peptides D102, isoD102, synthetic peptides D102, isoD102-spiked ADC-A digest, un-spiked ADC-A digest, and heat-stressed ADC-A digest.

A side-by-side RP–LC–MS analysis was performed with synthetic peptide D102, synthetic peptide isoD102, ADC-A digest (un-spiked), ADC-A digest spiked with D102 synthetic peptide, ADC-A digest spiked with isoD102 synthetic peptide, and digest of ADC-A stressed at 40 °C for 3 months. UV overlays are shown in Figure 4. Highly similar MS/MS mass spectra of the synthetic peptides D102, isoD102, and deamidated N102 in ADC-A digest are illustrated in Supplementary Figure S1. The difference in the elution times of synthetic D102 and isoD102 enabled the identification of deamidation degradants in ADC-A. As shown in Figure 4, the peak with D102 was only present in the synthetic peptide D102 and the D102 peptide-spiked ADC-A digest. In contrast, the peak with isoD102 was present in the synthetic isoD102, the un-spiked ADC-A digest, ADC-A digest spiked with synthetic isoD102, and the digest of heat-stressed ADC-A. These results demonstrate that the N102 in ADC-A deamidates solely to isoD102, which is unique, as generally the hydrolysis of the succinimide intermediate of N deamidation generates a mixture of isoD and D (Supplementary Figure S2).

**Figure 5:**
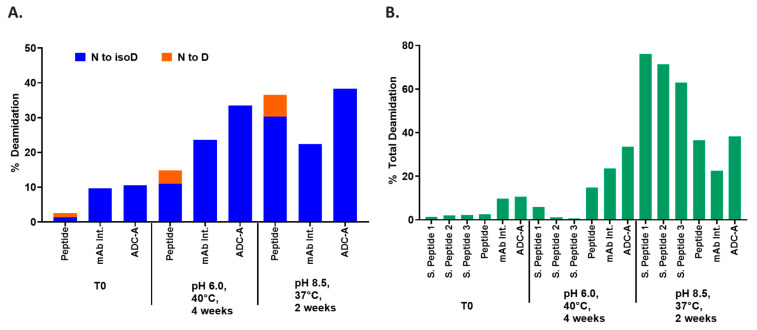
Comparison of NW deamidation products and deamidation rates in synthetic peptides, ADC-A, and mAb intermediate under heat stress and pH stress conditions. (**A**): NW deamidation level for each deamidation product; (**B**): total N102 deamidation level in synthetic peptides, ADC-A, and mAb intermediate.

Next, a heat stress study at pH 6 under 40 °C and at pH 8.5 under 37 °C was carried out to compare deamidation products from synthetic peptide N102, mAb intermediate, and ADC-A (Figure 5A). Under both pH environments, synthetic peptide N102 generated a mixture of isoD102 and D102, while ADC-A and its mAb intermediate only produced isoD102, thereby demonstrating that the higher order structure of the mAb intermediate and ADC-A play a role in preventing the formation of D102 during the hydrolysis of succinimide intermediate.

Factors affecting deamidation rate were also assessed within these heat stress/pH stress studies by comparing degradation levels of ADC-A, mAb intermediate, and synthetic peptide N102 to N102 short peptides 1, 2, and 3 (Figure 5B). Results show the following: (1) at both pH 6 and pH 8.5, NW deamidation rates were affected by the amino acid at n − 1 and n + 2, as well as the length of the peptides/proteins; (2) at pH 6, the deamidation rate of the N102 peptide was higher than the shorter peptides, but lower than the mAb intermediate and ADC-A, demonstrating that a higher order structure likely promotes NW deamidation; (3) the deamidation rate in ADC-A was even higher than in the mAb intermediate, indicating that conjugation in the hinge region could alter the micro-environment/local structure of N102, thereby making it more prone to deamidation; (4) at pH 8.5, shorter synthetic peptides deamidated to drastically higher levels than the full-length N102 peptide, the mAb intermediate, and the ADC-A, indicating that solvent exposure became the dominant factor affecting deamidation rate at a high pH.

### 3.4. N102 Conversion to isoD102 Impacts ADC-A Biological Activities More Than N102D Mutant

After discovering that N102 deamidates solely into isoD102, the impact of isoD102 versus D102 on ADC-A’s biological activities was investigated. First, ADC-A possessing D102 was generated via mutagenesis (i.e., an N102D mutant). The N102D mutant was then tested for binding and cytotoxicity alongside isoD102 (from CEX pre-peak 1), unmodified N102 (from CEX main peak), and the ADC-A Reference Standard (possesses ~15% deamidation).

The CEX profiles of unmodified N102, isoD102, and N102D mutant ADC-A are shown in Supplementary Figure S3. Peptide mapping verified that 100% of N102D mutant molecules contained the D mutation in both HC-CDRs, and that none of the mutated residues were of the isoD-form. Peptide mapping also measured 49% deamidation for isoD102, which corresponds to nearly all of the ADC-A molecules possessing isoD102 in one HC. Lastly, peptide mapping measured approximately 1% deamidation for unmodified N102, confirming the near absence of deamidation. Other tests assessing DAR, aggregates, and fragments by RP-LC, SEC, and CE-SDS, respectively, showed no other differences between isoD102, the N102D mutant, and unmodified N102 (Supplementary Table S2).

**Figure 6:**
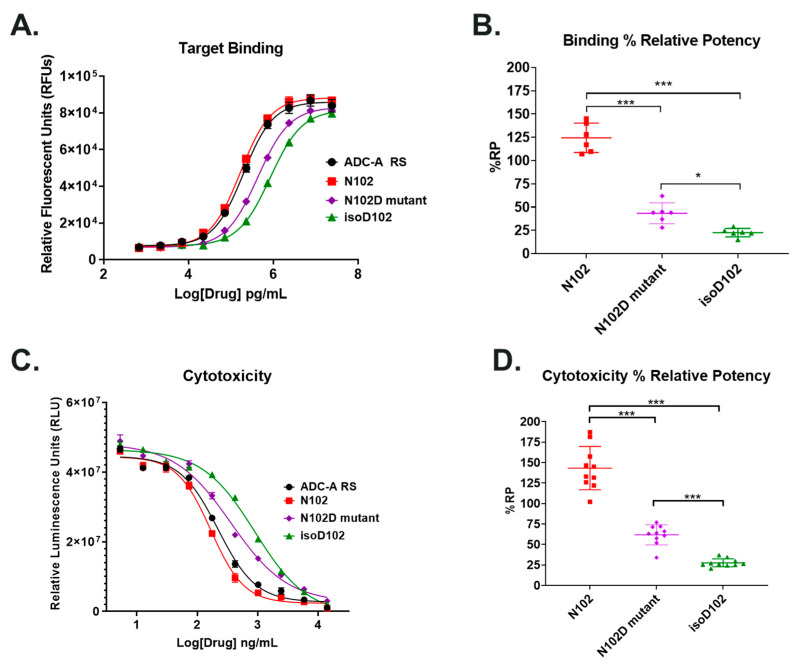
Comparison of binding and cytotoxicity between ADC-A Reference Standard (RS; black circles), N102 (red squares), N102D mutant (purple diamonds), and isoD102 (green triangles). Representative (**A**) binding and (**C**) cytotoxicity data are plotted as the mean ± SEM from a single assay run testing each sample in triplicates. Data were fit to a 4-parameter logistic (4PL) curve fit. Calculated % relative potency (%RP) for (**B**) binding and (**D**) cytotoxicity, as compared to ADC-A RS. Data are plotted from six assays runs for binding and ten assay runs for cytotoxicity, testing each sample in triplicates. Bars indicate the mean ± SD. Statistical significance was determined via a one-way ANOVA, with a post hoc Bonferroni’s multiple comparisons test (* *p* < 0.05, *** *p* < 0.001).

When tested for target antigen binding and cytotoxicity, the ADC-A N102, N102D mutant, isoD102, and Reference Standard showed comparable maximum effects, as demonstrated by the convergence of asymptotes across the 4PL curve fits in Figure 6A,C. However, these ADC-A samples show clear differences in potency for binding and cytotoxicity, as demonstrated by offsets in the 4PL curve fits across the x-axis, and thus varying EC_50_/IC_50_ values. Specifically, N102 ADC-A bound the target antigen with a mean %RP of 125%, compared to the ADC-A Reference Standard (Figure 6B). As the ADC-A Reference Standard possesses ~15% deamidation at N102, the slight increase in %RP of N102 ADC-A, which has little to no deamidation, is expected. In contrast, the N102D mutant binding was significantly reduced to a mean of 43% RP, relative to the Reference Standard. IsoD102 binding was further reduced to a mean of 23% RP, which was statistically less than both N102 and N102D mutant binding. Similar trends were observed for cytotoxicity, where N102, the N102D mutant, and isoD102 demonstrated mean %RPs of 143%, 62%, and 28%, respectively (Figure 6C). Similar to the binding results, isoD102′s further reduction in cytotoxicity %RP was statistically significant, compared to both unmodified N102 and the N102D mutant.

**Figure 7:**
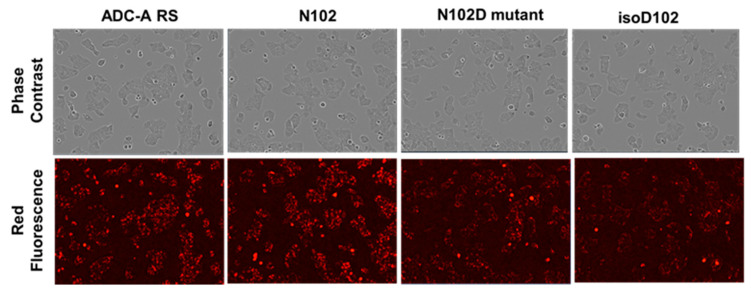
Example high-content fluorescent images following 5.5 h of incubation with 2 µg/mL ADC-A samples labeled with a pH-sensitive red fluorophore.

The cellular internalization of ADC-A RS, N102, N102D mutant, and isoD102 were compared in T47D cells via high-content fluorescent imaging. Internalization was detected by labeling ADC-A samples with a pH-sensitive red fluorophore, which only fluoresces in the low pH environment of a lysosome (see Section 2 for details). Images in Figure 7 show bright red fluorescence when cells were incubated with 2 µg/mL ADC-A RS and N102 for 5.5 h, indicating robust internalization. In contrast, incubation with N102D mutant and isoD102 showed a qualitatively dimmer fluorescence after 5.5 h of incubation, indicating less internalization. Phase contrast images showed consistent confluency and morphology when incubated with ADC-A samples, implying consistent cell health across treatments and that the cytotoxicity of the warhead had yet to take effect, following 5.5 h of incubation.

**Figure 8:**
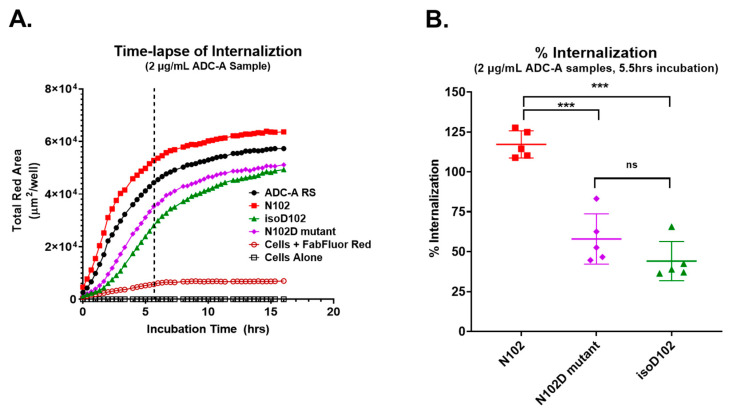
Quantitation of ADC-A internalization in T47D cells. (**A**) Time-course of ADC-A sample (2 µg/mL) internalization. Data points are plotted as the mean from four images per well at each timepoint. Background fluorescence was measured by incubating cells with FabFluor Red only (open red circles), as well as cells alone (open black squares). The dashed vertical line designates 5.5 h of incubation. (**B**) Percent internalization was determined relative to ADC-A RS at the 5.5 h timepoint. Data are plotted from five assay runs. Bars indicate the mean ± SD. Statistical significance was determined via a one-way ANOVA, and a post hoc Bonferroni’s multiple comparisons test (*** *p* < 0.001).

Red fluorescence was quantified as the total fluorescent area within each assay well and then plotted over time (Figure 8A). The data show that ADC-A RS and N102 internalized faster and at a higher saturation level, compared to the N102D mutant and isoD102. The % internalization of each ADC-A sample was calculated relative to ADC-A RS after 5.5 h of incubation and plotted side-by-side, as shown in Figure 8B. These data demonstrate that N102 internalized more than both the N102D mutant and isoD102. The N102D mutant showed a trend of more internalization than isoD102; however, this trend was not statistically significant.

### 3.5. Hydrogen–Deuterium Exchange Analysis of the N-Form and the isoD-Form of ADC-A

To investigate the impact of N102 deamidation on mAb intermediate/ADC-A protein structure, which might subsequently lead to bioactivity loss, HDX–MS was performed with mAb intermediate N102, isoD102, and the N102D mutant. Specifically, deuterium uptake (D-uptake) comparisons were made between N102 and isoD102 and between N102 and the N102D mutant, to reveal any conformational differences between the isoforms. To achieve the best results, HDX experiments required proteolytic digestion to yield enough common peptides from all the protein forms and to localize the comparison of conformational differences between N102, isoD102, and N102D mutant. The sequence coverage for N102 and isoD102 comparison was 82.9% for HC with 3.0 average redundancy, and 70.6% for light chain (LC) with 1.7 average redundancy. For N102 and N102D mutant comparison, HC sequence coverage was 91.6% with 4.0 redundancy, and LC coverage was 93.0% with 3.6 redundancy.

The HDX data acquired for the N102 molecule at different times and on different instruments were comparable. In particular, the HC-CDR1, as well as N- and C- termini, exhibited high D-uptake. Hinge peptides containing cysteines had poor sequence coverage due to an inefficient reduction in disulfide bonds at low temperature, but the region near the hinge, which is structurally dynamic, exhibited a relatively high D-uptake.

In many cases, residues in the HC-CDR3 region are responsible for target binding [23,24,25,26,27]. Consistent with this, the mAb intermediate HC-CDR3 is very rich in aromatic residues, and thus very likely to bind the target antigen through hydrophobic interactions generated by these residues. HDX data show relatively low D-uptake in mAb intermediate HC-CDR3, indicating it is structurally stable and therefore likely to be important for target antigen binding.

**Figure 9:**
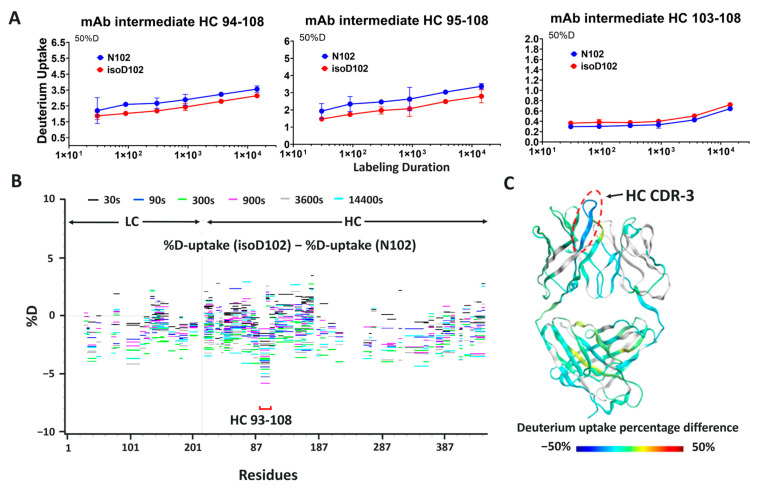
HDX comparison of the mAb intermediate in the N102 form and the isoD102 form. (**A**) D-uptake plots (measurements in triplicates) indicated that peptides in heavy chain CDR-3 were impacted by the deamidation at N102. The isoD102 form (red) compared with the N102 form (blue) showed D-uptake reduction in heavy chain CDR-3 peptides, suggesting that the local structure around N102 was altered upon deamidation. (**B**) HDX differential “Woods” plot of mAb intermediate light and heavy chains showed the difference in D-uptake between the N102 and isoD102 forms. Each line representing the peptide across the sequences (x-axis) is color coded with the HDX time points. The vertical position of each line on the y-axis indicates the level of D-uptake difference observed for this peptide. Lines below “0” are peptides with decreased D-uptake upon deamidation. Only peptides in HC-CDR3 showed consistent decreases in D-uptake. (**C**) Fab region of the mAb intermediate homology model color-coded with the HDX results. Green-colored regions represent no difference between the N102 and isoD102 forms. Blue-colored region indicates decreased D-uptake in HC-CDR3.

When comparing the D-uptake of mAb intermediate N102 to that of isoD102, no significant difference was observed globally. However, when focused on peptides containing the heavy chain 102 residue, reduced D-uptake was observed for isoD102 (Figure 9A). Specifically, isoD102 peptides 94–108 and 95–108 show 10–20% decreases in D-uptake compared to the same N102 peptides. In contrast, peptide 103–108, which lacks N102, shows minimal difference between the two isoforms. The Woods plot in Figure 9B indicates that the D-uptake differences in other peptides are all within 5%, suggesting no global structural changes were induced by the deamidation reaction.

**Figure 10:**
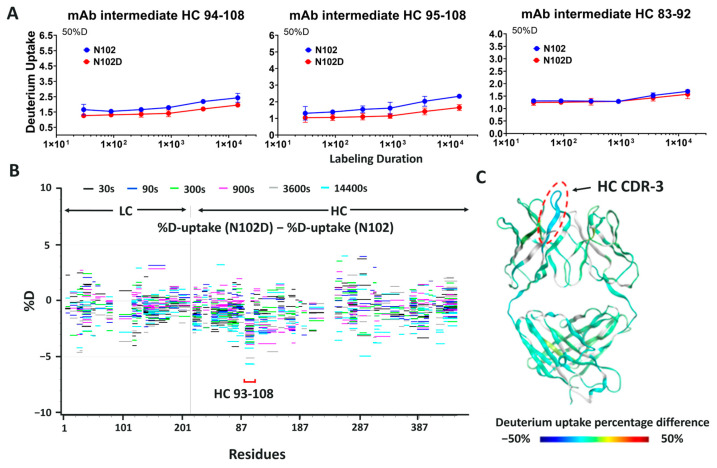
HDX comparison of the mAb intermediate in the N102 form and the N102D form. (**A**) D-uptake profiles (measurements in triplicates) of peptides in heavy chain CDR-3 exhibited lower D-uptake in the N102D form. The N102D form (red) compared with the N102 form (blue) showed D-uptake reduction in heavy chain CDR-3 peptides similar with the D-uptake decreases observed in the isoD102 form. (**B**) HDX “Woods” plot of mAb intermediate light and heavy chains showed D-uptake differences between the N102 and N102D forms. D-uptake decreases in HC-CDR3 were less prominent than in the isoD102 form (**C**) Fab region of the mAb intermediate homology model color-coded with the HDX results. Green-colored regions showed no difference between the N102 and N102D forms. Only HC-CDR3 was showing D-uptake decrease, and therefore is colored blue. However, the level of decrease observed was lower than in the isoD102 form.

Similar results were observed for the comparison between the N102 and N102D mutant. Again, peptides containing the 102 residue exhibited lower D-uptake in the N102D mutant (Figure 10A), implying less amide hydrogen is solvent accessible. However, the differences in D-uptake observed between N102 and isoD102 is greater than the differences between the N102 and N102D mutant (Figure 9B versus Figure 10B and Figure 9C versus Figure 10C), suggesting that the deamidation reaction forming isoD102 has a greater impact on the structure of adjacent residues than the N102D mutation. Together, these results suggest no global conformational change was induced by the single site modification at N102, whether through deamidation or mutagenesis. However, the local structure was impacted by N102 deamidation into isoD102 or N102 mutation to D102, in which isoD102 demonstrated greater conformational change.

### 3.6. Modeling the Structure of N102, isoD102, and the N102D Mutant

To enable visualization of the conformational changes, D-uptake differences are color-coded in the homology model of the mAb intermediate (Figure 9C and Figure 10C). Blue regions in the figure indicate decreased D-uptake, suggesting that amide hydrogen is less solvent accessible in N102D and isoD102. Gray color regions have no sequence coverage, and green indicates no difference in the comparison. In the three-dimensional structure shown in Figure 9C and Figure 10C, the only difference of significance observed is within the heavy chain CDR-3, which exhibits reduced solvent accessibility upon deamidation to the isoD102 form and mutation to N102D. Furthermore, reduced solvent accessibility is more pronounced in the isoD102 than in the N102D mutation, as indicated by the darker blue color in Figure 9C than Figure 10C.

**Figure 11:**
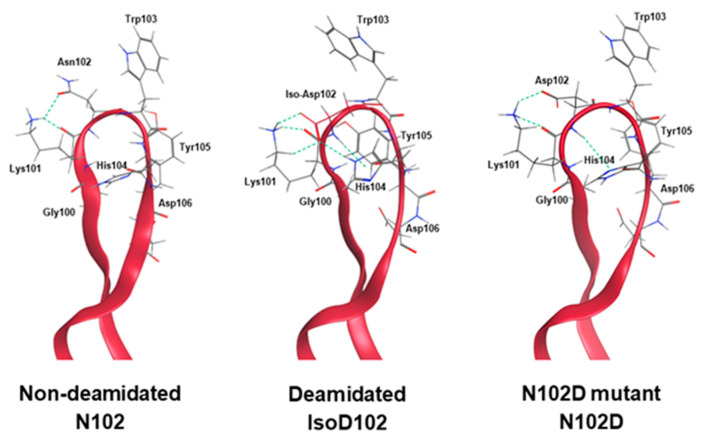
HC-CDR3 in N102, isoD102, and N102 homology models. Residues 100–106 were displayed, with hydrogen bonding indicated by the dotted lines. Compared with the N102D form, the isoD102 sidechain is stabilized by two additional hydrogen bonds, and therefore a more favorable form with lower free energy.

A molecular operating environment (MOE) was used to further model the protein structure at the amino acid level (Figure 11). MOE predicted the hydrogen bonding around the deamidation/mutation site for N102, isoD102, and N102D, with energy minimization in the CDRs. Typically, deamidation removes an ammonia group and replaces it with a water group on the sidechain of asparagine residues. However, in this case, deamidation caused an isoD102 formation in which the sidechain of isoD102 has an additional CH2 group inserted into the peptide backbone, thereby causing greater conformational changes. This change orients the side chain of the isoD102 closer to the adjacent K101. The nucleophilic side chain of the K101 residue then stabilizes the isoD102 side chain through the formation of two hydrogen bonds. Interestingly, modeling of the N102D mutant shows only one hydrogen bond with the adjacent K101, implying that the increased hydrogen bonding with K101 might make isoD102 more stable and more thermodynamically favorable compared to D102. The model also predicts that the amide hydrogen of isoD102 or D102 residue will form N–H π hydrogen bonding, which provides a valid explanation for the decreases in D-uptake observed in isoD102 and N102D forms, as the amide hydrogen of isoD102/D102 is engaged in hydrogen bonding and less solvent accessible. As a result of these local structural changes, adjacent residues may alter their side chain orientations, which could impact target binding. For example, the side chain of Y105 extends towards the outside in N102 form, but its position changes towards the inside of CDR-3 in the N102D mutant, and even further inside for isoD102. Also notably, the orientation of W103 and H104 are minimally altered when comparing the N102D mutant to N102, whereas their orientations are significantly flipped in isoD102.

## 4. Discussion

Previous studies report that the NW amino acid motif is commonly found within the CDRs of biotherapeutic antibodies, but that this motif possesses a low propensity to deamidate [12,13,20]. In contrast to these reports, we discovered a labile N102–W103 motif within the CDR-3 of ADC-A and its mAb intermediate, which is readily deamidated. The observed NW deamidation is increased under normal manufacturing and process conditions, as well as under thermal stress. Consistent with the deamidation of other motifs [10,11,12,13], the observed NW deamidation within the ADC-A/mAb intermediate is influenced by primary and higher-order structures, as evident from studies that have used synthetic peptides.

To elaborate, thermal stress increases the deamidation of both ADC-A and its mAb intermediate, which is not influenced by the pH of the solvent (Figure 5A). In contrast, a synthetic peptide composed of the NW motif and with an identical sequence to the native ADC-A/mAb intermediate deamidates to significantly higher levels when in a basic solvent, compared to an acidic solvent (Figure 5A). The deamidation’s dependency on the solvent pH is magnified when the peptide is shortened, in which deamidation levels reach < 10% at pH 6.0 versus > 60% at pH 8.5 (Figure 5B; S. Peptide 1). Altogether, these results imply that NW deamidation becomes less dependent on solvent exposure as the peptide chain lengthens and becomes less flexible, and as the complexity of the surrounding structure increases. Thus, the deamidation of N102–W103 within ADC-A and its mAb intermediate appears predominantly influenced by higher-order protein structure.

Experiments with synthetic peptides also demonstrate that residues contiguous to the NW motif influence its deamidation, at least when the motif is solvent exposed. Specifically, S. Peptide 2 (H to V substitution at n + 2) deamidated to a lower level than S. Peptide 1 under both acidic and basic environments (Figure 5B). S. Peptide 3 (K to V substituted at n − 1) deamidated to even lower levels under both environments (Figure 5B). Future studies are needed to determine whether deamidation of NW is equally influenced by these contiguous residues, when contained within the higher-order structure of ADC-A and its mAb intermediate.

Another contributing factor to the liability of this NW motif, when contained within a full antibody, could be the presence of the inserted cysteines in the HCs, which again act as the site of payload conjugation near the hinge region to make the ADC-A [21,22]. These inserted mutations may perturb the otherwise stable NW micro-environment, rendering it more susceptible to deamidation. Furthermore, the addition of a bulky and hydrophobic linker-warhead to these sites might further perturb the NW micro-environment, thereby explaining why the N102 deamidation rate is more rapid in ADC-A than in its mAb intermediate, under all pH- and thermal-stress conditions (Figure 1, Figure 2 and Figure 5).

Further studies are needed to understand how the inserted cysteines and/or payload conjugation within the hinge region of ADC-A/mAb intermediate influence the CDR-3 3D micro-environment to enable NW deamidation. So far, preliminary data continue to support the hypothesis that the NW deamidation, when contained within the full antibody, is reliant on hinge engineering/conjugation. Specifically, we produced a recombinant Fab that has an identical sequence to the Fab in ADC-A or its mAb intermediate (verified by peptide mapping). The produced Fab and the CEX pre-peak 1 (fraction from ADC-A with 49% N102 deamidation) were incubated at pH 9 under 40 °C for 1 day and then 25 °C for a week. We then performed peptide mapping on the stressed recombinant Fab and pre-peak 1. As expected, the N102 deamidation in the pH-/thermal-stressed pre-peak 1 continued to increase from 49% to 87%. Surprisingly, the N102 in the pH-/thermal-stressed recombinant Fab was nearly unchanged, resulting in only a 1.1% increase in deamidation (Supplementary Figures S4 and S5). UV chromatograms matched with the mass spectrometry data, in that only native N102 peak was observed in the pH-/thermal-stressed recombinant Fab—the deamidated N102 peak was not found. In contrast, the majority of the native N102 peak was deamidated into two isoD102 peaks (one from complete digestion, and one from incomplete digestion) in the stressed pre-peak 1.

So far, these data confirm that, without the hinge region and thus the inserted cysteines and payload conjugation, the NW motif within CDR-3 Fab is resistant to deamidation. Likewise, the payload conjugation of ADC-A also impacted N325-K326 motif in the Fc region. As shown in Figure 1A,B, N325 deamidation was 14% in the 40 °C one-month-stressed mAb intermediate, as is typically observed in monoclonal antibodies [19,28]. However, N325 deamidation was significantly lower (8%) in ADC-A under the same stress condition, implying conjugation had a protective effect against deamidation in this case. Additional studies are ongoing to further understand the influence of the hinge region’s cysteine insertion and payload conjugation on these deamidation sites, which will be addressed in a future publication.

N102 within the ADC-A/mAb intermediate was shown to deamidate solely into iso-aspartate (Figure 4), which is atypical [2,3,4]. As previously mentioned, deamidation of asparagine could happen via the three degradation pathways: (1) nucleophilic attack of the alpha-NH group on the carbonyl carbon of the side chain, leading to a five-membered succinimide (cyclic imide) intermediate that hydrolyzes into a mixture of aspartate and iso-aspartate; (2) nucleophilic attack by the backbone carbonyl oxygen on the carbonyl carbon of the side chain, resulting in a cyclic isoimide intermediate, yielding only aspartate after hydrolysis; or (3) asparagine residues deamidating to aspartic acid only by direct water-assisted hydrolysis [2,12,20]. As degradation pathway (1) above is the only pathway to result in iso-aspartate, it is logical to conclude that N102 within ADC-A/mAb intermediate solely deamidates through the formation of succinimide intermediate.

Results within Figure 5A demonstrate that the NW motif itself can deamidate into a typical mixture of iso-aspartate and aspartate, at least when the motif is contained within a simple peptide and the deamidation demonstrates solvent dependencies. However, deamidation of N102–W103 continued to convert solely into iso-aspartate within ADC-A and mAb intermediate, independent of the solvent pH. Thus, we conclude that this phenomenon is not inherent to the NW motif itself, but instead is induced by the unique higher-order structure surrounding N102–W103 within the ADC-A/mAb intermediate. Structural modeling confirmed that the conversion of N102 to isoD102 is thermodynamically favored over D102 in this antibody, as it results in additional hydrogen bonding with the adjacent K101 as opposed to D102 (Figure 11). In addition, the conversion to isoD102 appears to have a greater impact on the micro-environment structure compared to D102, as evident by both the modeling in Figure 11 and HDX data in Figure 9 versus Figure 10.

Both isoD102 and the N102D mutant impacted the biological activity of ADC-A in vitro. This impact stems from a reduced antigen binding affinity, which consequentially reduces ADC-A’s rate of cellular internalization, delivery of payload, and ultimately cytotoxicity. The reduced biological activities of isoD102 were more severe than the N102D mutant. In fact, the reduction in isoD102′s bioactivity is even greater than the apparent reductions in Figure 6, Figure 7 and Figure 8, as the isoD102 used in these experiments (i.e., from CEX pre-peak 1) likely possessed deamidation at only one Fab arm of ADC-A, whereas the N102D mutant possessed the intended mutation at both Fab arms. IsoD102 ADC-A from the CEX pre-peak 2, which likely possesses deamidation at both Fab arms, could not be used for biological characterization due to limitations in material amounts.

The HDX data in Figure 9 and Figure 10 show that isoD102 induces greater changes to local structure than N102D, which generally explains why isoD102 impacted biological activity more than the N102D mutant. MOE modeling provides some insight into the specific structural changes that may be occurring under isoD102 and the N102D mutant. Specifically, the conversion of N102 to isoD102 alters the orientation of the residues W103, H104, and Y105 more severely than conversion to N102D (Figure 11), which correlates with the severity of the reduction in biological activity in vitro. Altogether, these results demonstrate that N102 within the ADC-A/mAb intermediate solely deamidates into iso-aspartate because it is thermodynamically favored, which in turn induces greater changes to the local CDR-3 structure, and, thus, impacts biological activities more severely.

The impact of N102 deamidation on ADC-A’s in vivo activities has not been studied yet. However, we hypothesize that the profound reduction in ADC-A’s in vitro biological activities reported here will translate to reduced potency and/or efficacy for inhibiting tumor cell growth in vivo. N102 deamidation could also impact ADC-A’s pharmacokinetic properties, as cellular internalization and subsequent degradation of an ADC is not only integral to its mechanism of action, but is a major route to its metabolism, too [29]. Thus, reduced on-target cellular internalization could translate to increased systemic circulation of ADC-A in vivo, which may in turn increase the chances of off-target toxicities. Future studies are needed to fully assess whether N102 deamidation impacts these in vivo activities, and to test whether the differential impact between isoD102 and N102D mutant on in vitro activity is recapitulated in vivo.

Likewise, future studies are needed to determine the impact of N102 deamidation on the stability of ADC-A and its mAb intermediate as a drug product. While we show that accelerated stability under thermal stress increases N102 deamidation (Figure 1, Figure 2 and Figure 5), we have not yet studied how N102 deamidation feeds back to influence the overall stability of ADC-A and its mAb intermediate under accelerated and/or normal manufacturing and storage conditions. Previous studies report that asparagine deamidation can affect antibody colloidal stability and increase susceptibility to aggregation, especially under acidic conditions [30,31]. Considering these reports, it is possible that N102 deamidation will decrease the overall stability of ADC-A and its mAb intermediate, and that this instability will be more severe when N102 is converted to isoD102 versus N102D. Future studies are needed to detail this impact.

In summary, this is the first report on an atypical increase in asparagine deamidation in the non-canonical NW motif within an ADC and its mAb intermediate, which converts the asparagine residue solely into iso-aspartate. The observed NW motif is located within a site critical for antigen binding, which consequently impacts all other biological activities required for ADC-A’s mechanism of action. Interestingly, the conversion of the asparagine residue into iso-aspartate impacts biological activities more severely than conversion into aspartate, which correlates with HDX and modeling data showing more severe structural changes when the asparagine is converted to iso-aspartate. The unique susceptibility of the otherwise stable, non-canonical NW motif within the ADC-A/mAb intermediate is likely due to a distinct higher-order structure and specific susceptibilities in the N102 micro-environment brought on by the hinge region, including the inserted cysteines and/or conjugation of payload [21,22]. Future studies are needed to fully elucidate the mechanism of this NW deamidation, as well as to evaluate its impact on in vivo activity and influence on drug product stability. Overall, these findings help in assessing sequence liabilities, as well as ADC designs and constructs, when engineering and manufacturing antibody-drug conjugates and other mAb-based biotherapeutics.

## Figures and Tables

**Table 1:** Deamidation levels in CEX fractions by peptide mapping.

Sample Description	Deamidation (%)	
	N102	N389, N394
Starting Material (DS)	10	5.4
Main Peak	1.6	6.2
Pre-peak 1	49	7.2
Pre-peak 2	65	7.5

**Table 2:** Synthetic peptide sequence information.

Synthetic Peptide	Sequence
N102	(R)XXX**KNWH**XXXXXXXXXXXXXXXXXXXXK
D102	(R)XXX**KDWH**XXXXXXXXXXXXXXXXXXXXK
isoD102	(R)XXX**KisoDWH**XXXXXXXXXXXXXXXXXXXXK
S. peptide 1	(R)XXX**KNWH**X
S. peptide 2	(R)XXX**KNWV**X
S. peptide 3	(R)XXX**VNWH**X

## Data Availability

The data used to support the findings of this study can be made available by the corresponding author upon request.

## Supplementary Materials

The following supporting information can be downloaded at: https://www.mdpi.com/article/10.3390/antib12040068/s1, Figure S1: Highly similar MS/MS from synthetic peptides with D102, isoD102, and deamidated N102 in ADC-A tryptic digest; Figure S2: NW deamidation pathway in ADC-A and its mAb intermediate; Figure S3***:*** CEX profiles of ADC D-form (D102 in both HC), isoD-form (isoD102 in one HC, N102 in another HC) and N-form (N102 in both HC); Figure S4: Peptide mapping UV overlay of pH 9-stressed Recombinant Fab (top) and Fab that was generated via digestion from pH 9-stressed CEX Prepeak 1 (bottom), with native N102 peak and deamidated isoD102 peaks labeled; Figure S5: Zoomed-in Peptide mapping UV overlay of pH 9-stressed Recombinant Fab (top) and Fab that was generated via digestion from pH 9-stressed CEX Prepeak 1 (bottom), with native N102 peak and deamidated isoD102 peaks labeled. Table S1: Physicochemical testing of CEX fractions; Table S2: Physicochemical testing of N102, isoD102, and N102D Mutant. LOQ is defined as limit of quantification.
