# Supplementary material for: Atypical Asparagine Deamidation of NW Motif Significantly Attenuates the Biological Activities of an Antibody Drug Conjugate
Source: Antibodies (Basel). 2023 Oct 24;12(4):68. doi: 10.3390/antib12040068 (PMC10660493; doi:10.3390/antib12040068)

Supplemental Material

***Supplementary Table S1**:* Physicochemical testing of CEX fractions

Sample Description	DAR by Reduced RP-LC	HMW by SEC, %	LMWs by nrCE-SDS, %	LMWs by rCE-SDS, %	DAR by RP-LCMS	LMWs by RP-LCMS	HMW (covalent bond) by RP-LCMS
Start material	2.20	0.5	13.3	4.6	2.20	Comparable	Undetected
Main peak	2.16	0.7	13.2	4.6	2.16		
Pre-peak 1	2.14	1.8	14.4	5.7	2.14		
Pre-peak 2	Not Tested				comparable	6% ↑	

***Supplementary Table S2**:* Physicochemical testing of N102, isoD102, and N102D Mutant. LOQ is defined as limit of quantification.

Test		N102	isoD102	N102D Mutant
DAR		2.27	2.27	2.17
HPSEC	Monomer (%)	99.3	99.2	96.2
	Aggregate (%)	0.7	0.6	3.6
	Other/Fragment (%)	0.0	0.2	0.2
CE-SDS (non-reduced)	Purity (%)	86.0	86.7	87.3
	Monomer (%)	86.0	86.7	87.3
	Leading Peak (%)	4.2	3.9	4.6
	Shoulder (%)	0.0	0.0	0.0
	½ Antibody (%)	8.0	7.5	6.1
	Other (%)	1.9	1.8	1.9
CE-SDS (reduced)	Purity (%)	95.2	94.5	95.7
	Heavy Chain (%)	66.3	65.6	66.1
	Light Chain (%)	29.0	28.9	29.7
	HCLP (%)	1.8	1.9	1.0
	Fragment (%)	<LOQ	<LOQ	0.0
	Other (%)	2.9	3.5	3.3

Supplementary Figure S1: Highly similar MS/MS from synthetic peptides with D102, isoD102, and deamidated N102 in ADC-A tryptic digest.

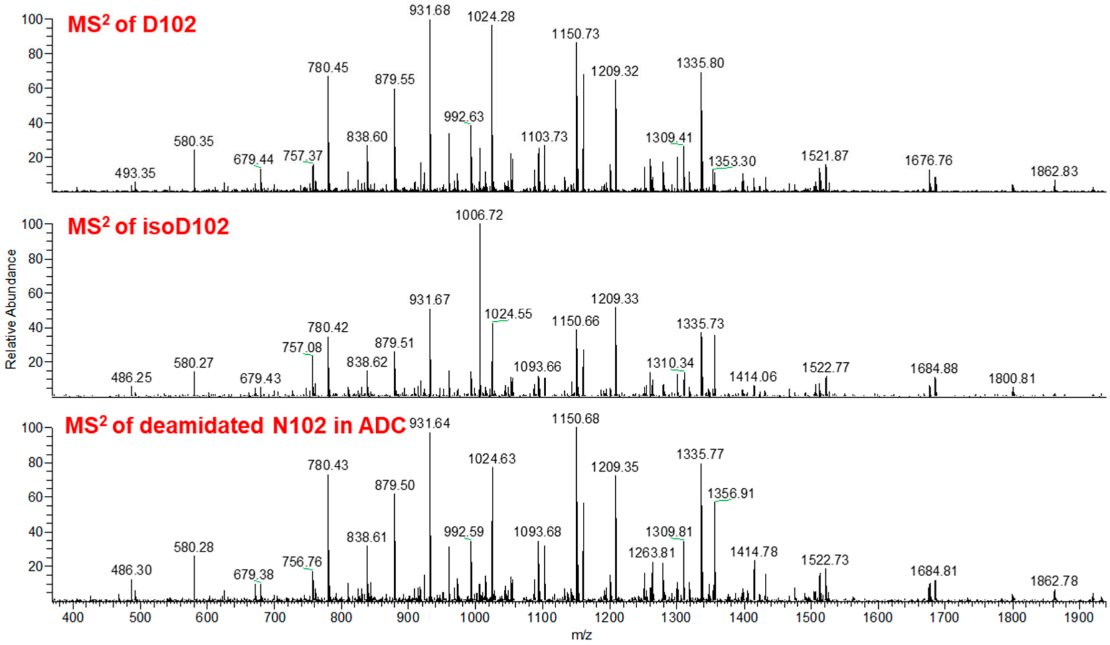

Supplementary Figure S2: NW deamidation pathway in ADC-A and its mAb intermediate. The red arrow designates the direction of the nucleophilic attack from the alpha-NH group on the carbonyl carbon of the side chain within the asparagine residue. The succinimide intermediate within ADC-A's NW deamidation pathway solely converts to iso-aspartic acid (illustrated with green check mark) and not aspartic acid (illustrated with red prohibition sign).

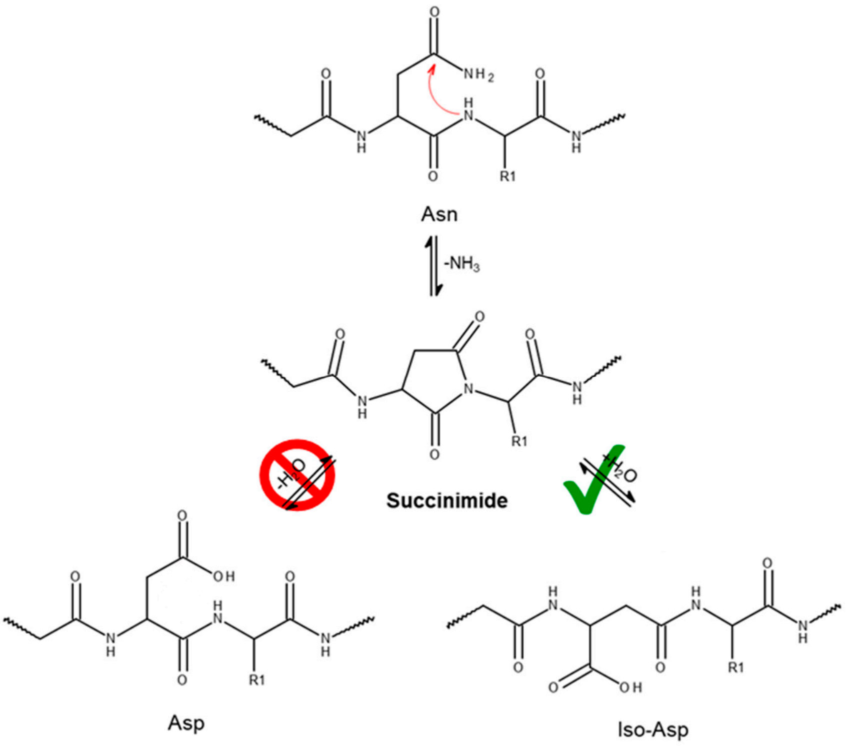

Supplementary Figure S3: CEX profiles of ADC D-form (D102 in both HC), isoD-form (isoD102 in one HC, N102 in another HC) and N-form (N102 in both HC)

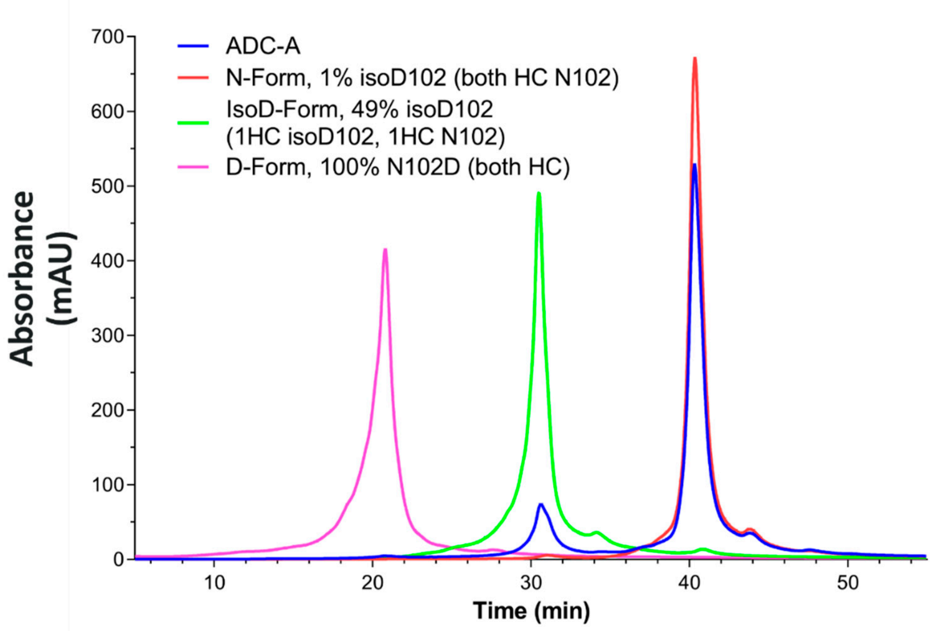

Supplementary Figure S4: Peptide mapping UV overlay of pH 9-stressed Recombinant Fab (top) and Fab that was generated via digestion from pH 9-stressed CEX Prepeak 1 (bottom), with native N102 peak and deamidated isoD102 peaks labeled.

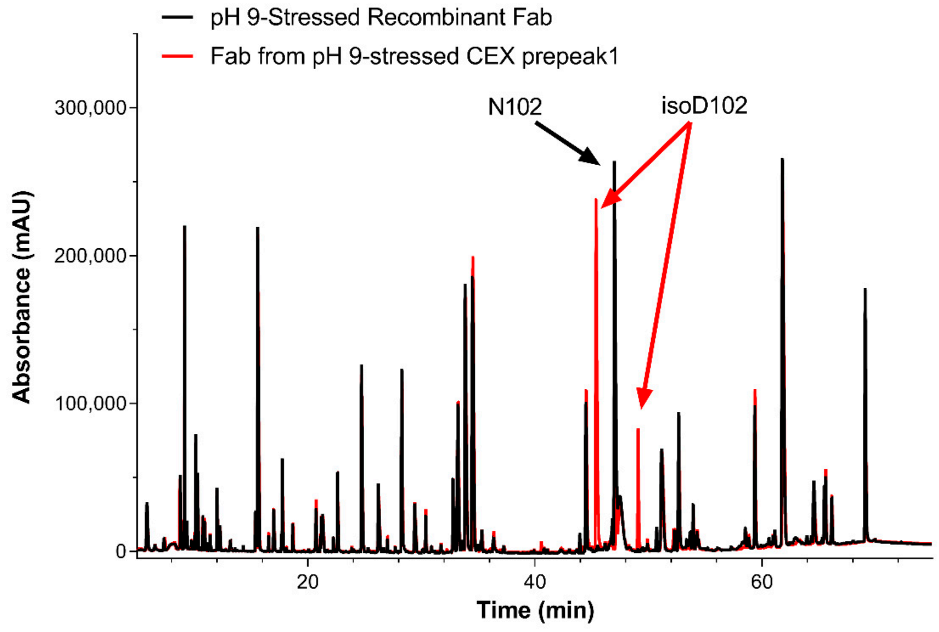

Supplementary Figure S5: Zoomed-in Peptide mapping UV overlay of pH 9-stressed Recombinant Fab (top) and Fab that was generated via digestion from pH 9-stressed CEX Prepeak 1 (bottom), with native N102 peak and deamidated isoD102 peaks labeled.

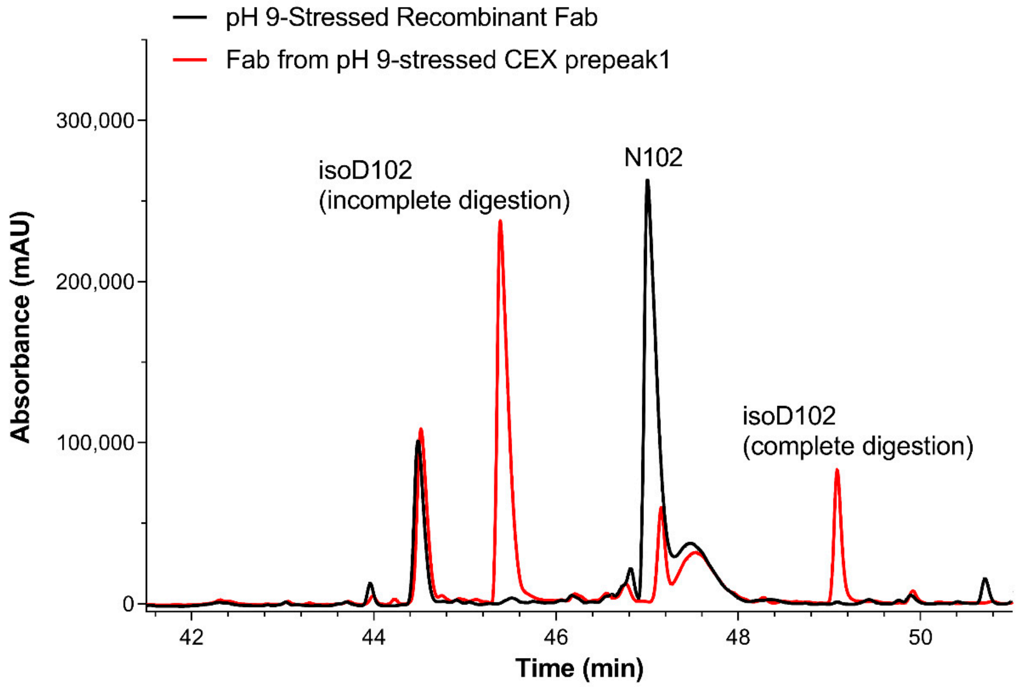

Supplement: Supplementary file 1 [file antibodies-12-00068-s001.zip › antibodies-2627148-supplementary.pdf]
